# Barriers, challenges, and facilitators in oral health care for older adults in home-based care: stakeholder perspectives from germany

**DOI:** 10.1186/s12877-026-08219-7

**Published:** 2026-09-10

**Authors:** Sarah Porzelt, Alena Koenig, Maren Heyke, Anja Behrens-Potratz, Peter Stratmeyer, Petra Schmage, Claudia Konnopka, Stefanie Schellhammer, Martin Scherer, Alexander Konnopka, Thomas Zimmermann, the InSEMaP group, Thomas Beikler, Marie Drecktrah, Paula Harmes, Espen Henken, Lydia von Palubitzki, Doniyor Yuldashev

**Affiliations:** 1 Department of General Practice and Primary Care, University Medical Center Hamburg-Eppendorf, Center for Psychosocial Medicine, Hamburg, Germany; 2 Department of Nursing and Management, Cooperative Process Management in Social and Healthcare RTC (KoPM-Zentrum), Hamburg University of Applied Sciences, Faculty of Business and Social Sciences, Hamburg, Germany; 3 Department of Periodontics, Preventive and Restorative Dentistry, University Medical Center Hamburg-Eppendorf, Center for Dental and Oral Medicine, Hamburg, Germany; 4 Department of Health Economics and Health Services, University Medical Center Hamburg-Eppendorf, Center for Psychosocial Medicine, Hamburg, Germany; 5 Department of Health Care Research and Innovation, Deutsche Angestellten Krankenkasse - Gesundheit (DAK-G), Hamburg, Germany; 6 Department of Psychiatry and Psychotherapy, University Medical Clinic Leipzig and Leipzig University, Leipzig, Germany

**Keywords:** Oral health care, Home-based care, Interprofessional collaboration, Older adults, Stakeholder perspectives, Dental service access, Germany, Care dependency, Long-term care

## Abstract

**Background:**

With the growing number of people requiring long-term care in Germany, not only is the prevalence of chronic diseases increasing, but oral health is also deteriorating. Many older adults with functional impairments who live at home can no longer maintain their oral hygiene independently and therefore rely on caregivers. Consequently, the quality of oral care often depends these caregivers. However, as oral health care in the outpatient setting involves multiple stakeholders — including dentists, dental assistants, general practitioners, professional and family caregivers- the fragmented German healthcare system, characterised by distinct responsibilities and reimbursement models, often results in poor coordination among those stakeholders.

We aimed to explore the perspectives of key stakeholders, including family caregivers, professional caregivers, dentists, dental assistants, and general practitioners, to identify barriers, challenges, and facilitators related to both the delivery and utilisation of oral health care for older adults in need of home-based care.

**Methods:**

As part of the ‘*Interaction of Systemic Morbidity and Oral Health in Ambulatory Patients in Need of Home Care (InSEMaP)*’ study, we conducted a descriptive qualitative study incorporating both homogeneous and heterogeneous stakeholder focus groups, complemented by individual interviews with professionals involved in oral health care for home-based care recipients in Hamburg, Germany. All interviews were digitally recorded, transcribed verbatim, and analysed using deductive-inductive qualitative content analysis following Kuckartz’s framework.

**Results:**

A total of 55 stakeholders participated in six homogeneous focus groups (n = 29), two heterogeneous focus groups (n = 9), and 17 individual interviews. Three main themes emerged: (1) *Facilitators and Challenges in Oral health care*, (2) *Utilisation of Dental Care*, and (3) *Interprofessional Collaboration*.

**Conclusions:**

Improving oral health care for older adults in need of home-based care requires greater awareness of its relevance to overall health and well-being. Our findings highlight the need for structured interprofessional collaboration, alongside reforms in reimbursement models, caregiver training, digitalisation, and coordination of care. Addressing these factors may contribute to improving oral health care services for older adults with reduced functional capabilities.

**Trial registration:**

DRKS00027020. Date of Registration: 04/01/2022; https://drks.de/search/de/trial/DRKS00027020.

**Supplementary Information:**

The online version contains supplementary material available at https://doi.org/10.1186/s12877-026-08219-7.

## Background

Due to demographic changes, particularly the aging population, a higher proportion of individuals with care needs can be expected in the future. In 2021, approximately five million people in Germany required care [1]. One-third of those requiring care are very old. The majority of care is provided at home: four out of five care-dependent people are cared for by relatives, with support from outpatient care services, or a combination of both. Just over half of these individuals receive only care allowance, indicating that they are generally cared for at home by family members. Around 20% of care-dependent individuals receive assistance from outpatient services, either alongside family care or instead of it. Only about 793,000 people (16%) are in full-time inpatient care in nursing homes [1].

In Germany, home-based care (HBC) is remunerated through the statutory long-term care insurance and includes both formal services by professional home care agencies and informal support from family or volunteers [2]. Professional caregivers—such as registered nurses or geriatric-qualified staff—visit patients’ homes to assist with activities of daily living (including basic oral hygiene), administer medications, perform wound care, monitor vital signs, and coordinate with General Practitioners (GP) and other professionals/therapists. They also handle documentation per long-term care insurance requirements and facilitate medical aids [3, 4].

GPs play a crucial role in overseeing and coordinating medical care for care-dependent older adults, ensuring continuity between outpatient and home-based services. Informal caregivers typically support daily routines when professional services are unavailable. Unlike inpatient or clinic-based care, home-based care aims to preserve the independence and quality of life of older adults in their familiar environment [5]. This blend of formal and informal support forms the framework in which oral health care for home-based care dependent older adults should be integrated [6].

In this manuscript, *oral health care utilisation* refers to the extent to which older adults in need of care make use of available oral health care measures, including both daily hygiene routines and professional dental services. *Access to dental services* refers specifically to the availability and usability of clinical dental treatments, including physical, financial, and organisational dimensions. *Home-based care* (HBC) describes care services provided in the patient’s private living environment, including support with oral hygiene as part of daily care. Where appropriate, we use the term *home-based oral health care* to describe the provision of oral health care within this setting. *Oral health care* (OHC) refers to all measures promoting and maintaining oral health, including both daily hygiene routines and clinical dental services. The term *dental services* is used more specifically for professional treatment provided by dentists and dental assistants, such as examinations, procedures, and home visits.

The ageing process is inherently associated with a higher prevalence of chronic and multiple diseases, often leading to care dependency among older adults [7, 8]. As the number of older adults receiving care grows, so does the prevalence of chronic conditions and deteriorating oral health [9–11]. This decline is driven by multiple factors: age-related changes, such as xerostomia caused by polypharmacy [12], functional impairments and cognitive deficits [13, 14] restrict effective daily hygiene. At the same time, structural barriers–such as immobility, financial constraints, and a lack of barrier-free dental practices – limit access to dental services [13, 15–17].

In the context of multimorbidity, OHC often becomes a low priority both for care recipients and within the healthcare system [10, 18, 19].

Older adults in need of HBC are especially vulnerable. Emerging evidence indicates that oral health is closely linked to systemic health [20, 21]; poor oral health has been associated with chronic diseases like diabetes and cardiovascular disease, as well as malnutrition and aspiration pneumonia-conditions particularly prevalent in older populations [10, 14, 20, 21]. These associations highlight the importance of maintaining oral health; however awareness of these interactions remains limited in routine care, further contributing to fragmented services [16, 22, 23].

Oral care by third parties is inherently complex; it extends beyond clinical technique to include interpersonal trust, feelings of shame, cultural values, caregivers’ willingness, and time availability–all of which influence the delivery of oral hygiene [16, 24].

Structural and contextual barriers contribute to a significant oral health gap between older adults in need of care and their non-care-dependent counterparts [16, 25].

In Germany, statutory and private health insurance generally cover basic dental services, with individuals often also purchasing supplementary insurance to extend coverage for dental services [26].

However, in practice, fragmented care structures obstruct the delivery of coordinated OHC for HBC patients. Stakeholders involved in home-based healthcare often lack awareness of each other’s roles, and pathways of care are poorly integrated. Furthermore, medical and dental care in Germany is organized in different sectors, each with its own self-administration, planning, and remuneration [26, 27].

To develop patient-centered OHC models that reflect the realities of HBC in Germany, it is essential to understand both the respective professional practices and the interprofessional interactions between them. This study aims to explore the perspectives of key German stakeholders—including family caregivers (FC), professional caregivers (PC), GPs, dentists, and dental assistants (DA)- involved in the outpatient care to adults aged 60 years and older. The goal is to identify barriers, challenges, and facilitators in improving OHC for older adults in need of HBC.

This investigation was part of the project titled *‘****In****teraction of ****S****yst****e****mic ****M****orbidity and Oral Health in ****a****mbulatory ****P****atients in Need of Home Care (****InSEMaP****)”* [27]. The InSEMaP target population is defined as older adults aged 60 years and older receiving HBC, as determined by the German Social Code (SGB XI). While the present study does not include this population directly, it explores the perspectives of stakeholders involved in their oral health care provision.

## Aim

This study aimed to identify challenges, barriers, and facilitators affecting the delivery and utilisation of OHC and dental services for older adults in need of HBC in Germany, from the perspectives of multiple key stakeholders. It also examined opportunities and limitations for interprofessional collaboration (IPC) in this context.

## Methods

### Research design

This study employed a qualitative exploratory design using three data collection formats: individual interviews, homogeneous focus groups, and heterogeneous focus groups to investigate stakeholder perspectives on OHC among care-dependent older adults [28–30]. It was implemented as part of the InSEMaP project and took place in Hamburg, Germany, between October 2022 and May 2024.

The study was carried out in two consecutive phases. Phase 1 included individual interviews and homogeneous focus groups held separately with five key stakeholder groups: family caregivers (FC), professional caregivers (PC), dentists, dental assistants (DA), and general practitioners (GP). Phase 2 consisted of heterogeneous focus groups that brought together representatives from different stakeholder groups to jointly discuss interprofessional collaboration and shared responsibilities in OHC.

The two-phase design was conceptually grounded in the goal of capturing both within-group and interprofessional perspectives. Phase 1 formats (interviews and homogeneous focus groups) enabled participants to reflect on their own roles and experiences within their respective stakeholder group, whereas Phase 2 heterogeneous focus groups fostered cross-professional dialogue.

This sequential approach enabled us to first explore stakeholder-specific perspectives and then contrast them in a shared dialogue, which enhanced analytical depth and reflexivity.

The study design aimed to capture both intra-group experiences and cross-sectoral perspectives, enabling a comprehensive understanding of the barriers, facilitators, and coordination challenges in oral health care for recipients of HBC.

To support the two-phase structure, two different interview guides were developed and tailored to the objectives of each phase (see Data collection for details).

### Data collection

Focus groups encourage small groups to engage in discussions on a specific topic, stimulated by prompts such as visual materials or case vignettes. A key strength lies in their ability to generate shared knowledge, though group dynamics can also shape the discourse in unintended ways. To balance these effects and capture individual perspectives in greater depth, we complemented the group discussions with individual interviews [28, 31, 32].

The interview guides were developed following Helfferich’s SPSS principle, a four-step process of collecting, examining, sorting and subsuming topics, to combine structure with openess and ensure comparability and flexibility [33, 34]. A generic guide was used across all stakeholder groups in the first phase, supplemented by group-specific questions. This guide was employed in both focus groups and individual interviews to ensure thematic consistency. Each guide included case vignettes, main and follow-up questions, and checklists, and covered four key themes (see supplementary file 1):

- facilitating and inhibiting factors affecting the provision of OHC.
- facilitating and inhibiting factors affecting utilisation of dental services.
- current state of interprofessional collaboration.
- wishes and ideas for future collaboration and improvement of OHC.

The second interview guide focused exclusively on interprofessional collaboration based on the findings from Phase 1. This allowed us to deepen the exploration of professional roles, expectations, and coordination mechanisms across stakeholder groups, directly addressing the study’s second objective (see Supplementary File 2).

Each individual interview and focus group concluded with an open-ended question inviting participants to share their wishes and ideas for improving interprofessional collaboration and OHC. This approach enabled the inclusion of future-oriented perspectives across all stakeholder groups.

The focus group pretest included three professional caregivers. Based on their feedback, we revised key questions and adjusted the sequencing of discussion prompts. The finalised procedure followed a standardised protocol, including technical setup, participant introductions, and documentation [31, 35].

### Sampling

The study focused on stakeholders involved in the OHC of older adults in need of HBC, as defined in the InSEMaP project (see Background). We applied purposive sampling to recruit participants representing five stakeholder groups relevant to OHC for older adults in HBC: GP, dentists, DA, PC, and FC. The sampling strategy aimed to capture a diverse range of insights regarding professional background, care setting (e.g., outpatient vs. informal care), and experience with older adults. For FC, variation was sought in terms of closeness to the recipient of care and duration of caregiving.

**Fig. 1:**
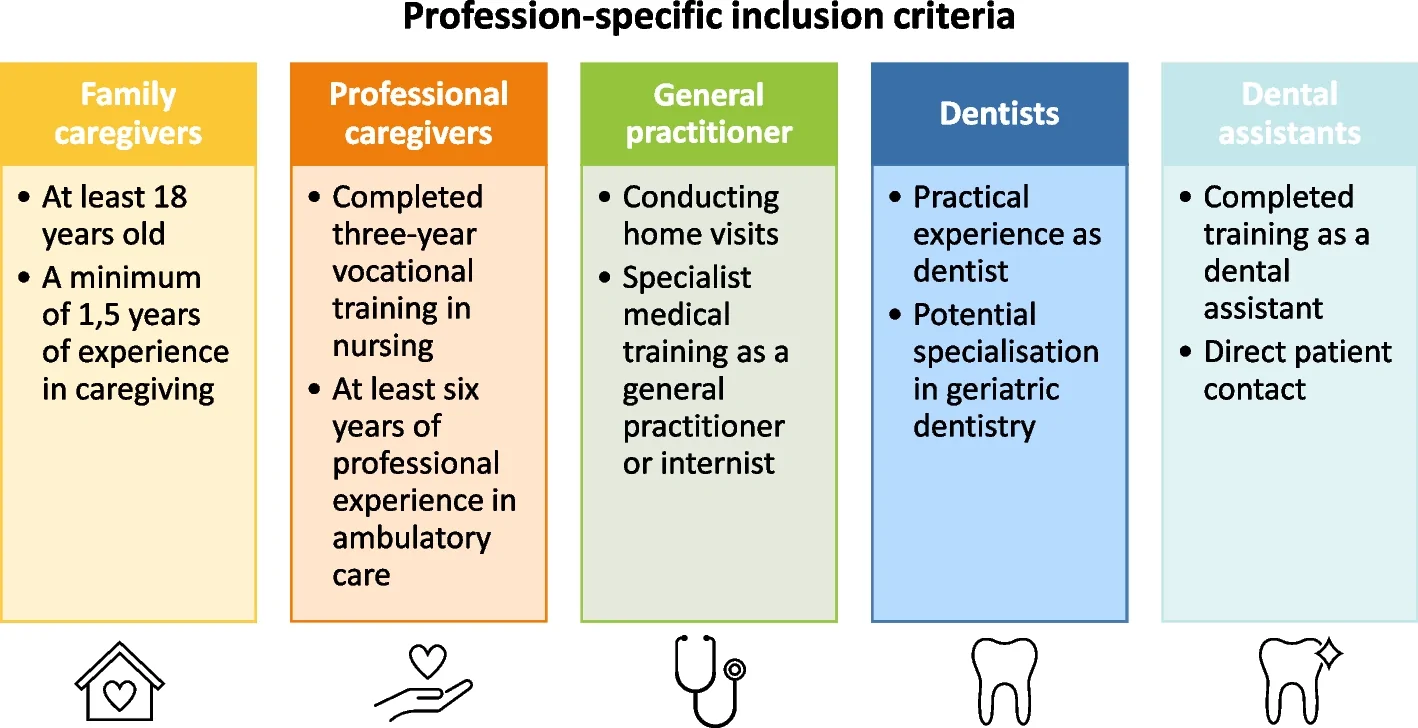
profession-specific inclusion criteria

For phase 1 (individual interviews and homogeneous focus groups), participants must have been actively providing oral health care or general care services to older adults in HBC at the time of data collection and thus were currently engaged in the OHC of older adults. General practitioners were included despite not being directly involved in HBC provision, as are often the primary medical contact and play an important role in coordinating care for older adults receiving HBC in Germany. Participants provided these services in Hamburg or the surrounding area and demonstrated essential German language skills. For phase 2 (heterogeneous focus groups), additional participants were recruited based on their active involvement in self-governing bodies, administration, or advocacy organisations related to HBC. No exclusion criteria were defined, as we aimed to be informed by a broad range of stakeholders. Other specialists (e.g. geriatricians) rarely conduct home visits in our region. All recruited GPs worked in community-based private practices, not in hospitals. Since there is no formal geriatric‐dentistry specialization in Germany, we recruited dentists who (1) regularly treat older, homebound patients (including conducting home visits) and/or (2) have completed at least one continuing‐education course on geriatric dental care. All participating dentists had provided care to older adults in a home‐based setting. Figure 1 provides an overview of the profession-specific inclusion criteria. While sample sizes were pre-defined based on the project design [27], we considered thematic saturation as an analytical reference point [36]. No new relevant themes emerged in the final Phase 1 individual interviews and focus groups within stakeholder groups, which suggests that key dimensions of the research questions were adequately captured. The sequential two-phase design also allowed us to validate and refine themes across different settings and stakeholder perspectives.

### Recruitment

Participants were recruited separately for each phase of data collection. For the first phase, participants for individual interviews were invited between October 2022 and March 2023, while stakeholders were recruited sequentially to ensure that focus groups could be conducted in close temporal proximity to the corresponding individual interviews. Data collection for the second phase took place between October 2023 and January 2024. Participant recruitment for both phases was carried out in cooperation with different stakeholder organisations in Hamburg. Since we aimed to capture perspectives within Hamburg care context, we limited recruitment to organisations based in Hamburg. This ensured that all participants operated within the same local care environment and allowed us to include diverse viewpoints from key organisations such as the Association of Statutory Dentists, the Association of General Practitioners, the Hamburg Society of Care, and the Alliance for Caregivers. Organisations were initially contacted by phone to confirm their willingness to support the recruitment process. Subsequently, they were provided with tailored recruitment letters to be distributed within the organisations. In the first phase of data collection these letters could be used either for internal distribution within the organisations or for directly approaching potential participants. Additionally, recruitment flyers were also created. Recruitment efforts primarily targeted participants for the homogeneous focus groups. Reminder emails were sent specifically to general practitioners approximately two weeks after the initial contact, as this group proved more difficult to recruit. No reminders were necessary for other stakeholder groups due to sufficient initial response rates.

However, when individuals were unable to attend the scheduled sessions, individual interviews were offered as an alternative. Participants for the second phase of data collection were predominantly contacted directly and were also provided with a recruitment letter.

Once individuals agreed to participate, they received detailed study information via email. This information included an overview of the InSEMaP project, the specific research methods (focus groups or interviews), and the role of the participants, who were asked to share their experiences regarding the OHC of older adults in need of HBC.

Additionally, data protection measures and the intended use of the collected data were explained. The document contained a consent form, which participants were required to sign and return prior to data collection. Furthermore, participants completed stakeholder-specific questionnaires capturing sociodemographic data and background characteristics of the sample.

Expense allowances of €40 for individual interviews and €100 for focus groups were available for all participants in both phases of data collection. Focus groups duration were planned to last approximately two hours, while individual interviews were scheduled for one hour.

### Ethics approval

The study adhered to the ethical principles outlined in the Declaration of Helsinki, ensuring the protection of vulnerable groups, such as older people and care-dependent older adults. Full transparency regarding the study, informed consent, and the anonymity and confidentiality of data were prioritised throughout the research process. The project received ethical approval from the Ethics Committee of the Hamburg Medical Association (2021–100715-BO-ff) on November 1, 2021. All documents, including recruitment materials, study information, consent forms, interview guides, and questionnaires, were submitted to the Ethics Committee for review and approval.

### Data collection

Focus groups were audio-recorded and documented via structured postscript protocols. Depending on participant group and accessibility, sessions were held either in person or conducted digitally. Cisco WebEx was used as a secure video conferencing tool that ensures confidentiality through password-protected, individual invitations. In-person sessions were arranged when digital formats posed a barrier to participation (e.g. among FC). The homogeneous focus group for FC was held in person to encourage participation.

Interviews were conducted either via telephone or in person, depending on participant preference. All interviews were audio-recorded with the participants’ consent and subsequently transcribed verbatim for analysis.

A baseline demographic form was used to collect data on participants’ age, gender, educational background and relevant experience. All focus group discussions were facilitated by AKoe or SP, with MH attending as a note-taker.

Data were collected by AKoe (four homogeneous focus groups, 13 individual interviews and two heterogeneous focus groups) and SP (two homogeneous focus groups, four individual interviews). Interviewers and participants were either unfamiliar or loosely acquainted, such as through professional associations. Prior to data collection, interviewers introduced themselves as project staff and provided both verbal and written information about the study. There were no dropouts between recruitment and data collection, and all participants gave written informed consent.

### Data analysis

Audio recordings were transcribed by research assistants following predefined transcription rules for computer-assisted analysis [33]. Transcripts were checked for accuracy by another assistant. The transcripts were not returned to participants for correction or comment. MAXQDA software was used for this process, allowing for synchronisation between audio and transcript, enabling researchers to re-listen to the recordings during analysis. Data were pseudonymised according to a protocol [33].

Data were analysed SP, AKoe and MH using the content-structuring method as described by Kuckartz [33], utilising both deductive categories from the interview guide and inductive categories emerging from the material. Main categories were defined a priori, while subcategories were generated during the analysis. All codes were based on meaningful units, ensuring that each segment was comprehensible on its own. After coding, categories were refined through internal discussions with research colleagues.

We ensured trustworthiness by applying established quality criteria for qualitative content analysis [33]. To enhance credibility, a subset of transcripts was coded independently by two researchers and discussed until consensus was reached. The coding system was developed iteratively and documented systematically. Triangulation across stakeholder groups and data sources (individual interviews and focus groups) supported analytical depth. Transferability was strengthened through detailed contextual descriptions and sampling transparency. An audit trail and reflexive memo-writing contributed to analytical rigour.

### Researcher characteristics

Researchers’ characteristics, beliefs and assumptions can influence qualitative research and data interpretation [36]. SP: female, research assistant, PhD student PhD, nursing science/care management (M.Sc.), experience in qualitative research (QR) in the field of health services/care research; AKoe: female, research assistant, postgraduate student of Public Health (MPH), experience in QR; MH: female, research assistant, postgraduate student of Health Economics and Health Care Management (M.Sc.), initial experience in QR. SP and AKoe were experienced in qualitative methods, and MH, who was new to qualitative research, received extensive training and close supervision.

## Results

A total of N = 55 participants took part in the study across two phases of data collection. In the first phase, 46 participants contributed to six homogeneous focus groups (n = 29) and 17 individual interviews. One focus group was held with each stakeholder group (FC, PC, GP, dentists and DA); for PC, two groups were conducted due to heterogeneity in roles. The first PC group included only individuals in management roles, while the second consisted of staff from a large outpatient care organisation.

In the second phase, nine participants took part in two heterogeneous focus groups, bringing together representatives from different stakeholder groups. Of the eight focus groups, six were conducted digitally via Cisco WebEx and two in person (the FC group and one of the PC groups). All 17 individual interviews were audio-recorded, of which 16 were conducted by telephone and one in person at the participant's request.

The age of participants ranged from 24 to 80 years (mean: 49.8 years). The mean duration of focus groups was 97 min (range: 75–121), and individual interviews averaged 36 min (range: 17–57). See Table 1 for sample characteristics.

**Table 1:**
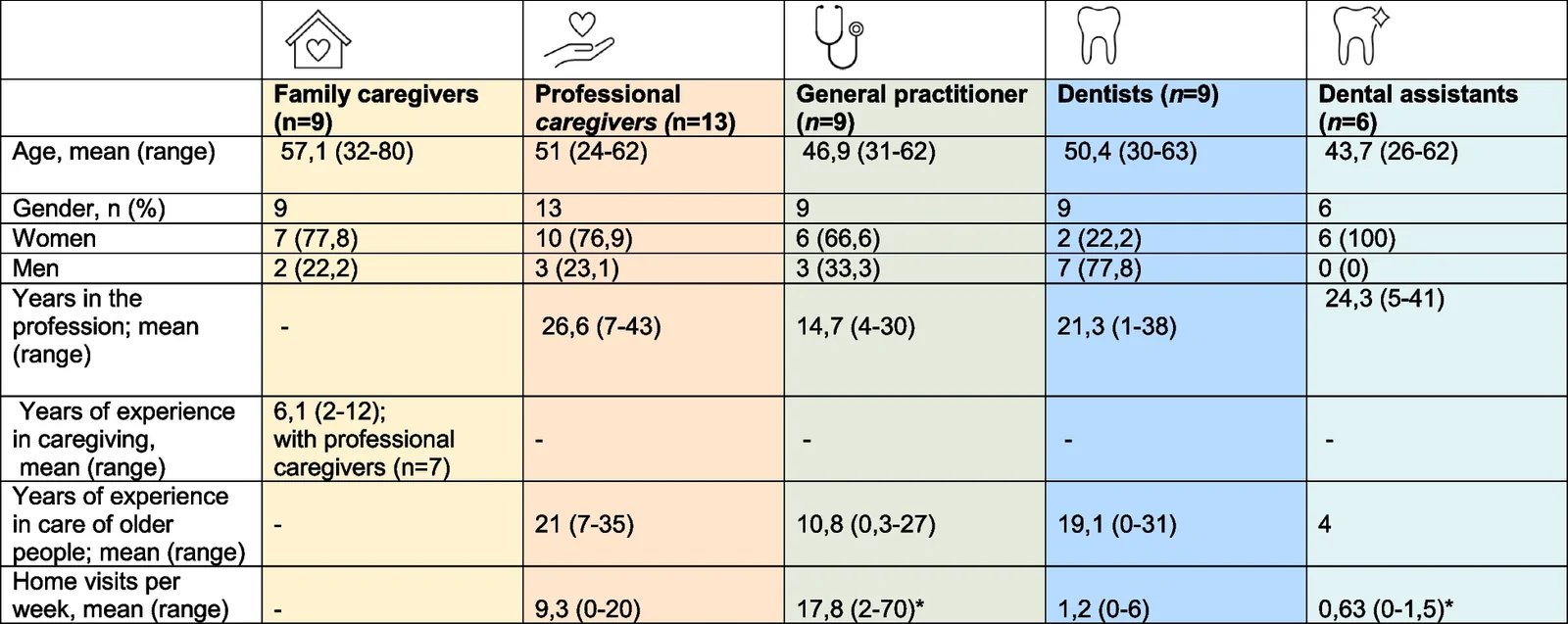
Characteristics of the participants (individual interviews and homogeneous focus groups)

*includes nursing home visits

The age of participants ranged from 24 to 80 years (mean: 49.8 years). The mean duration of focus groups was 97 min (range: 75–121), and individual interviews averaged 36 min (range: 17–57). See Table 1 for sample characteristics.

The analyses resulted in a coding framework that incorporated the perspectives of all stakeholder groups involved in Phase 1 of data collection (see Table 2). Four main categories were identified: Oral health care (OHC), Utilisation of dental care, Interprofessional collaboration, and Future perspectives. Within the first two categories, both facilitators and challenges emerged and are presented below. While barriers were more frequently discussed, facilitators are highlighted within each domain to illustrate supportive conditions for maintaining oral health in care-dependent older adults.

**Table 2:** Category system derived from qualitative content analysis across all stakeholder groups

Main category	Top category	Subcategory
**Oral health care**	Facilitators	Care network General description
	Challenges	Dementia/cognitive limitations Immobility/physical limitations Challenging behavior Shame Lower priority Time required/lack of time Lack of order Dentures Care network
**Utilisation of dental care**	Affecting factors	Facilitators Challenges
**Interprofessional collaboration**	Facilitators	Referrals/prescriptions Good accessibility Personal acquaintance Continuity Proximity/Network Regular exchange
	Challenges	Hierarchy Unclear responsibilities Lack of knowledge Lack of communication Different expectations Difficult to reach No personal knowledge Overload/too much effort Priority dentistry Data protection Financing
**Future Perspectives**		Framework conditions Education Communication

Table 3 provides a structured overview of the most salient themes and includes illustrative participant quotes to exemplify key findings. This thematic structure resulted from coding and analysis; not all themes are illustrated by quotes in this table.

**Table 3:** Summary of key themes with illustrative quotes

Main theme	Key Subthemes (condensed)	Illustrative Quote/Insight (condensed)
**Oral Health Care**		
**Facilitators**	– Supportive guidance or complete takeover of dental care by PC – Instruction on dental and denture care from DA to the FC and/or PC – Functioning care network	‘[…] *We also try to give some guidance to the family members or the care staff*.’ (DA, EI_ZFA06) ‘*Well, if/when people have those who actually come regularly to provide care, then I would say that it significantly improves*.” (GP, EI_GP09)
**Challenges**	– Dementia-related refusal and apraxia – Physical limitations – Time constraints for PC – Shame and intimacy – Lack of prioritisation	‘*It is often problematic to carry out tasks in what is […] an intimate area like the oral cavity.”* (PC, EI_PC06) ‘*… if we find a way to do that and still manage to get by over time and also be able to invoice it in that way, I think that will be difficult*.’ (FG3_PC, Pos. 80, Speaker: PC)
**Utilisation of dental care**		
**Facilitators**	– Functioning care network – Pain-driven treatment – Accessibility to dental practices/home visits – Financial incentives (health insurance Bonus booklet) – Recall systems of dental practices	*‘(…) they are reliant on other people, and that’s where the chain breaks down. As long as the patient themselves or their family members remember, it works. But once it’s handed over to someone else, it doesn’t work. (…)”* (FG5_ZÄ, Pos. 100, Speaker: Dentist) ‘*The predecessor used to do home visits occasionally, and we have continued that practice. And yes, we’ve actually expanded it. More and more patients have been asking for it, including those who are not regular patients at the practice, as well as patients who used to be cared for by the former dentists, but those dentists no longer offer this service.’* (FG5_ZÄ, Pos. 8, Speakers: Dentist 08/09) *‘(…) So, certainly, there are still a small number of patients who regularly come to us for check-ups. But often, you experience this with many patients who later become dependent on care: they mainly come in for pain treatment. And then, of course, you always try to intervene again and say, 'Well, let's do a thorough cleaning, get everything nice and clean again.' So, you try to intervene a little bit here and there. But with many, you actually find that they mainly come in for pain treatment afterwards. (…)*’ (EI_DA06, Pos. 56; Speaker: DA)
**Barriers**	– Physical and cognitive limitation – Lack of home visits/No possibility of access to the dental practice – Lack of care network – Shame or embarrassment – Financial burden – Lack of perceived need – Prioritisation challenges	*‘(…) and they come with someone, two or three people. And, yes, they come rarely. I would say. They don’t come often. Not even just for a check-up. I think the effort is probably too much for them.’* (FG4_ZÄ, Pos. 93, Speaker: Dentist03) '*(…) so, no need. There was no obvious issue. And if there isn't an immediate problem now, then honestly, I have enough on my plate already with organising everything else. (…)*' (EI_PA02, Position 66; Speaker: PA02)
**Interprofessional collaboration**		
**Facilitators**	– Geographic proximity & personal familiarity – Referral forms – Documentation tools – Structured communication	*‘So, we often still use the referral form because it ensures that at least the GP is informed about what happened. This is the only reason we do it, because we often get additional information from the general practitioner about what else should be done.’* (FG2_PP, Pos. 128, speaker: PP04- PC) *‘We have this documentation form, and I’ve found that sometimes you just have to take the initiative, like visiting the GP on a Wednesday afternoon or another day, and say, 'Okay, we have the following concept, and if you have patients of ours, I’d ask you to fill this out for our documentation.' And when you’ve done this with them a few times, it surprisingly works better and better.’* (FG5_ZÄ, Pos. 142, Speaker: ZÄ06- Dentist)
**Challenges**	– Lack of clear responsibilities – Hierarchies – Fragmented communication – Patients as intermediaries – Deprioritisation of oral health	*‘Up until that point when the dentist said, ‘Yes, we can’t manage this anymore”, and, well, I had to accept it.’* (EI_PA07, Pos. 122; speaker: FC) *‘We have so many things to take care of; teeth are important, but not that important. Then there’s mashed food—that works too. It’s not ideal for the person being cared for, but it’s just how society is now.’* (FG2_PP, Pos. 74; Speaker: PC- PP02)
**Future Perspectives**		
**Framework conditions**	– Better funding and reimbursement – Cooperation agreements – Integration in disease management programmes	*‘We usually bill through service packages, and the requirements set by the expert standard—not just that one, but other expert standards as well—actually call for a dedicated service package for oral hygiene in billing.’* (FG3_PP, Pos. 464, Speaker: PP12-PC)
**Education**	– Interprofessional education – Curricula expansion – Awareness materials for professional and family caregivers	*‘Training specifically for oral health is probably needed in all professional groups, except maybe for dentists—they already know this. But for all other groups, training on how important it really is, because I notice myself that I don’t deal with it much in everyday life either, since there’s always so much else going on.’* (EI_HÄ09, Pos. 110; Speaker: GP)
**Communication**	– Electronic patient records – Secure communication systems – Local care networks – Regular patient conferences and interprofessional case discussions	*‘I wish we all had the same communication system, let’s say in email form, where you could quickly write a short two- or three-liner, like 'I was here today, I noticed this and that,' or 'Everything is fine.' That’s what I would wish for. I really want to be the central point of contact as a general practitioner and know everything that’s going on.’* (EI_HÄ01, Pos. 130; Speaker: GP)

### Facilitators and challenges in OHC

This main category captures factors that either support or hinder daily oral hygiene and denture care among older adults receiving HBC, as described by all stakeholder groups.

### Facilitators in OHC

Stakeholders emphasized that many older adults tried to maintain independence in managing daily oral hygiene and denture care, or received guidance or support from PC or FC. PC regularly monitored oral hygiene and intervened early when potential problems arose. Among older adults with higher care needs, such as with higher level of care dependency, cognitive impairments, or dementia, oral care was often delegated entirely to caregivers, with FC and PC assuming a supportive or advisory role.

DA also contributed by advising on appropriate tools and techniques: *‘[…] we always try to get the patients to come for regular check-ups, and of course, we also try to make it possible for them to maintain their oral hygiene themselves, by providing them with aids that are feasible for them. We also try to give some guidance to the family members or the care staff.’* (EI_ZFA06, Pos. 18; speaker: DA).

A well-functioning care network between PC and FC was repeatedly highlighted as essential for maintaining oral health: *‘Well, if/when people have those who actually come regularly to provide care, then I would say that it significantly improves.’* (EI_GP09, Pos. 18; Speaker: GP).

### Challenges in OHC

Barriers to adequate OHC were frequently reported, particularly among older adults with dementia. PC and FC described difficulties due to loss of routine, resistance to care, and cognitive decline: *‘[…] there is, for instance, the client group affected by dementia. In such cases, it is often problematic to carry out tasks in what is, one could say, an intimate area like the oral cavity. Considerable persuasion is often required, and there are instances of apraxia, where they no longer understand the purpose of a toothbrush or similar things. […]’* (EI_PC06, Pos. 4; Speaker: PC).

Physical limitations also hindered daily oral hygiene and required adapted tools or additional assistance. Challenging behaviours, particularly refusal of oral care, posed major difficulties for both PC and FC, often leading to inadequate OHC. Feelings of shame further complicated care, particularly when trust was lacking.

A low prioritisation of oral hygiene in daily routines was linked to increasing care dependency and limited resources. PC stressed that time pressure and unclear responsibilities restricted the provision of adequate oral care: *‘ (…) if we find a way to do that and still manage to get by over time and also be able to invoice it in that way, I think that will be difficult.’* (FG3_PC, Pos. 80, Speaker: PC).

PC noted the absence of a clear care mandate or protocol for oral care within professional caregiving frameworks. This structural gap led to inconsistent or insufficient support, particularly when care recipients refused care or perceived it as unnecessary.

Denture-related issues such as poor fit or inadequate cleaning created further complications, often resulting in oral inflammation and infections. Overall, participants agreed that tailored and coordinated support was essential for maintaining oral health in this vulnerable group.

### Facilitators and barriers in utilisation of dental care

This main category encompasses influencing access to and utilisation of professional dental services by older adults in HBC. The most prominent themes included the functioning of care networks, physical and cognitive limitations, and structural aspects of service delivery (see Table 4).

**Table 4:**
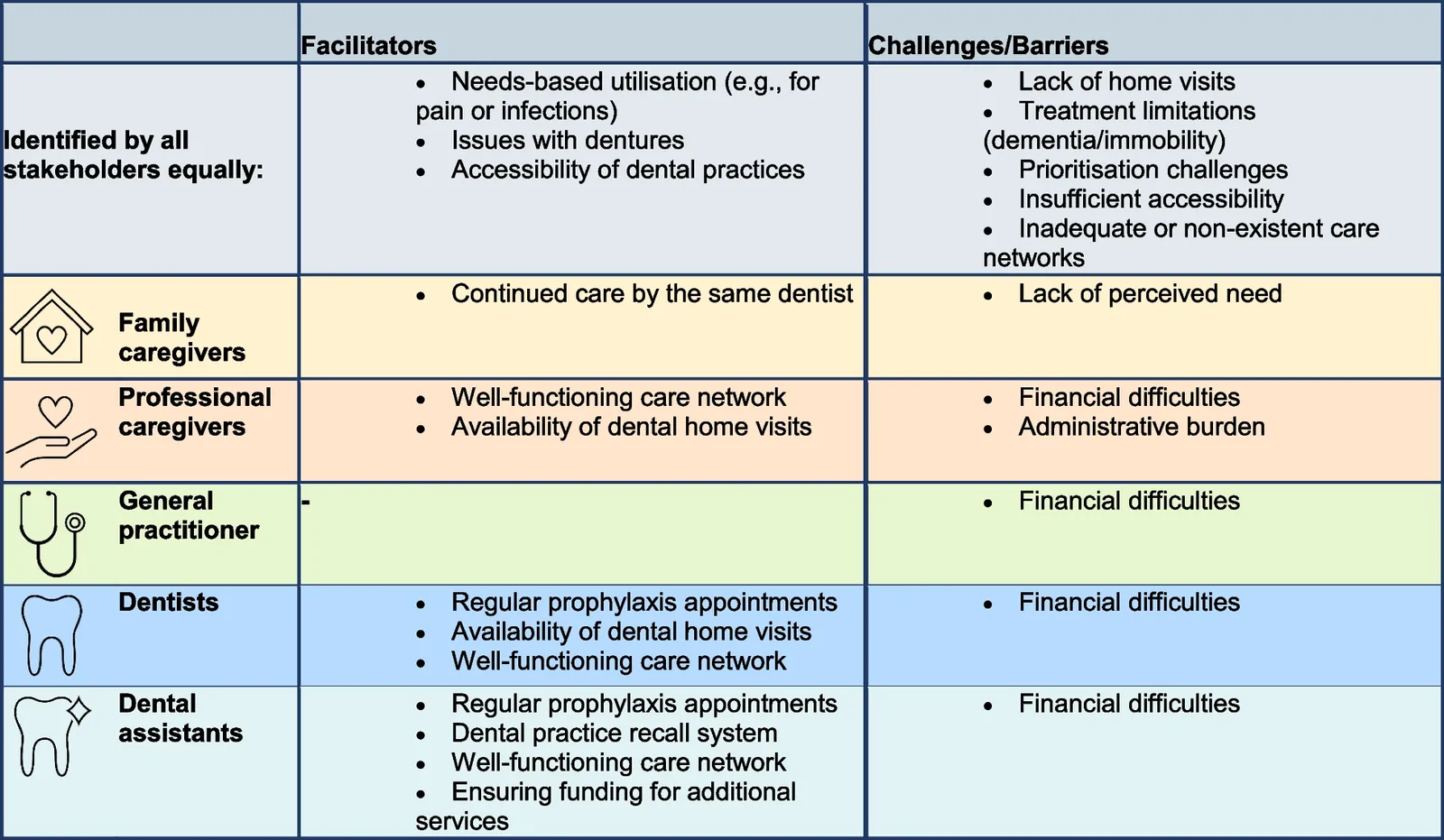
Barriers, challenges and facilitators of utilisation of dental care

### Facilitators of utilisation of dental care

A well-functioning care network, typically involving FC and/or PC, was viewed as crucial for scheduling and coordinating appointments, ensuring continuity of care, and promoting adherence to dental check-ups.

Conversely, a lack of coordination often impeded care, especially when responsibilities were unclear: *‘They are reliant on other people, and that’s where the chain breaks down. […] once it’s handed over to someone else, it doesn’t work. […]’* (FG5_ZÄ, Pos. 100, Speaker: Dentist).

Accessibility of dental practices and the availability of home visits further supported utilisation. In some cases, financial incentives such as the bonus booklet offered by statutory health insurance enhanced motivation for regular preventive visits.

However, stakeholders noted that preventive appointments were often replaced by problem-driven visits, typically triggered by acute pain or denture-related issues: *‘[…] many patients […] mainly come in for pain treatment afterwards. (…)’* (EI_DA06, Pos. 56; speaker: DA).

### Challenges and barriers to utilisation of dental care

Physical and cognitive impairments frequently hindered access to dental practices or positioning in the dental chair. In addition, transportation to dental practices posed both practical and financial challenges for some care recipients, particularly when home visits were unavailable and transportation costs were not reimbursed. Home visits, while beneficial, were rarely offered and were sometimes declined by patients out of shame. Coordination by FC was inconsistent, as other medical appointments were prioritised: *‘[…] They don’t come often. […] I think the effort is probably too much for them.”* (FG4_ZÄ, Pos. 93, Speaker: Dentist03).

Financial constraints further limited access, especially for professional dental cleanings that exceeded patients’ financial means. Some FC also expressed concern that dental procedures involving anaesthesia or sedation might exacerbate cognitive impairment: '*[…] if there isn't an immediate problem now, then honestly, I have enough on my plate already with organising everything else. […]*' (EI_PA02, Pos. 66; Speaker: PA02).

In summary, the utilisation of dental services was supported by coordinated care networks and accessible service structures but remained constrained by physical, cognitive, financial, and organisational barriers.

While barriers dominated stakeholder narratives, facilitators appeared across both domains and underscored the importance of maintaining supportive care networks, clear professional responsibilities, and accessible service structures to promote oral health among care-dependent older adults.

### Interprofessional Collaboration (IPC)

**Fig. 2:**
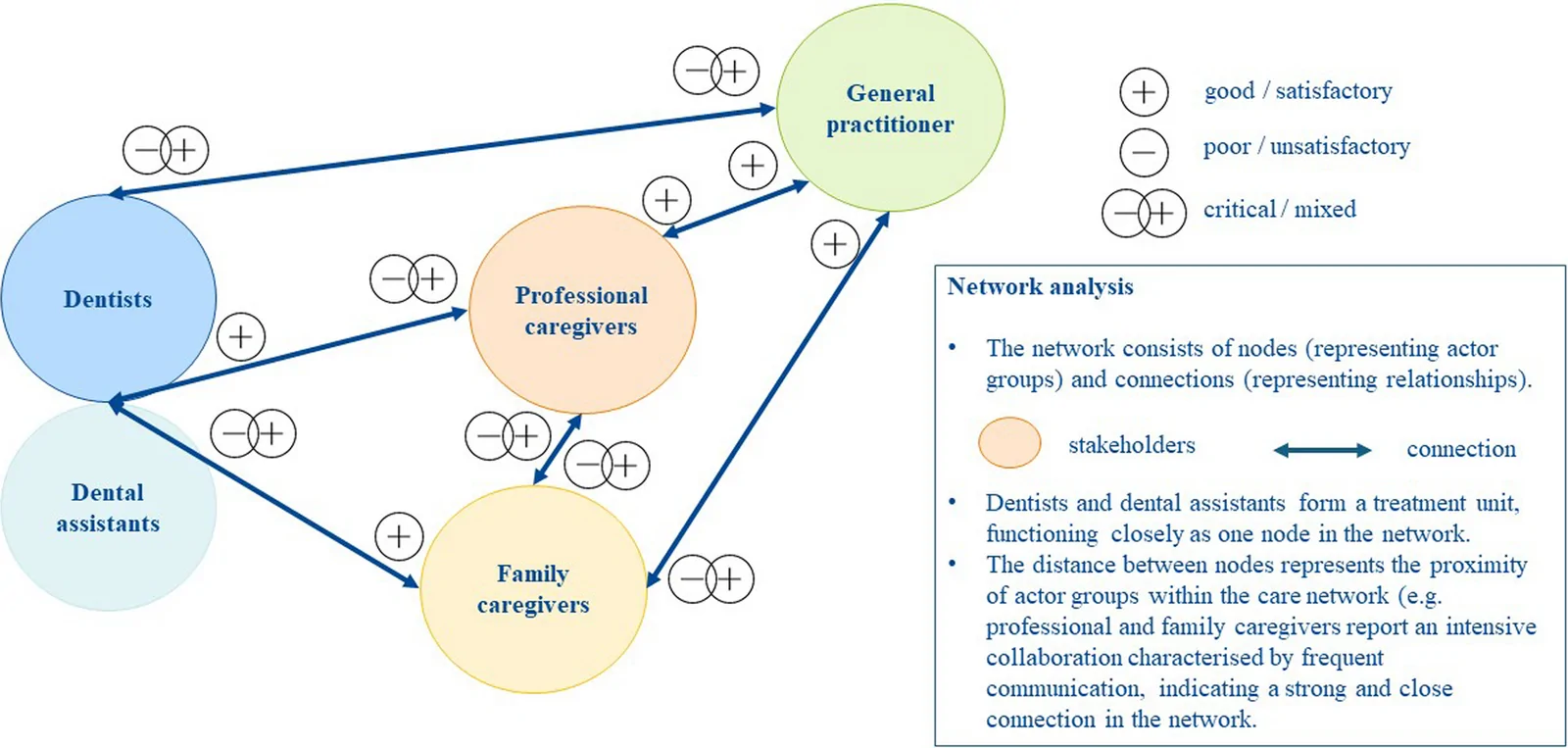
Interprofessional collaboration network of stakeholders in oral health care

This category examines experiences and perceptions of collaboration among stakeholders involved in OHC for older adults in need of HBC. It includes factors that facilitate or impede IPC. The IPC network of older people in need of HBC is illustrated in Fig. 2.

Professional caregivers reported mixed interactions with dental practices, often mediated through DA. General practitioners described collaboration as ranging from effective to minimal, while FC generally reported positive experiences with dentists.

All groups, except PC, had difficulties reaching GPs, who in turn perceived all stakeholders as hard to contact. FC reported challenges in connecting to dental services. Dentists and DA described varying levels of cooperation with FC and PC, shaped by differing expectations. General practitioners rated collaboration with PC as mostly positive due to structured processes, while PC had mixed experiences, often linked to prescription issues.

Stakeholders described varying communication pathways: PC mainly used indirect channels such as fax or email, particularly when contacting GPs. GPs and dentists relied primarily on fax, as reaching colleagues by phone was often difficult. Direct phone communication between professionals occurred occasionally, mainly between GPs or dentists and FC, while DA continued to use fax most frequently. Overall, patients or relatives often acted as intermediaries, bridging information gaps between professionals. The most common topics of IPC were medication inquiries and prescriptions.

### Facilitators of IPC

Stakeholders emphasised that IPC was enhanced by geographic proximity, personal familiarity, and continuity of care. GPs also noted that collaboration with FC intensified when they took responsibility for managing their relatives’ healthcare due to increasing care needs or dementia.

PC and GPs highlighted structured communication pathways, particularly concerning prescriptions and changes in health status.

While overall communication between stakeholders was often fragmented, some FC described situations where short and direct communication channels with PCS helped to coordinate care and exchange essential information. These examples referred mainly to urgent or routine issues within established care relationships.

Tools such as referral forms and documentation templates were reported to strengthen communication: *‘So, we often still use the referral form because it ensures that at least the GP is informed […].’* (FG2_PP, Pos. 128, speaker: PP04- PC) and *‘[…] sometimes you just have to take the initiative […], like visiting the GP on a Wednesday afternoon or another day […].’* (FG5_ZÄ, Pos. 142, speaker: ZÄ06- Dentist).

### Challenges to interprofessional collaboration

All stakeholder groups noted limited communication, often restricted to prescriptions or medication plans. FC frequently felt uninformed about other professionals involved, while PC reported fragmented structures and divided responsibilities, particularly regarding dental practices: *‘We have so many things to take care of; teeth are important, but not that important. […].’* (FG2_PP, Pos. 74; speaker: PP02-PC).

This lack of clear responsibilities, hierarchical barriers, and differing expectations further impeded collaboration. Dentists and DA reported low awareness of the association between oral and systemic health among GPs and PC. PC noted that FC frequently delegated the coordination of dental appointments due to work commitments. Conversely, FC described PC as overburdened, which was perceived to have an negative impact on OHC. Communication tools such as fax were considered outdated, and patients or relatives often had to act as intermediaries—resulting in information loss.

Both GPs and PC described difficulties in reaching each other, while FC found it unclear whom to contact for dental issues. Some FC expressed feeling left alone with coordinating dental appointments: *‘Up until that point when the dentist said, “Yes, we can’t manage this anymore”, and, well, I had to accept it.’* (EI_PA07, Pos. 122; speaker: FC).

Overall, stakeholders acknowledged that IPC in OHC for older adults was limited and mainly focused on medication and prescription issues. Nevertheless, they also offered concrete ideas on how to improve collaboration, service structures, and education. These proposals are summarised in the following section on Future perspectives.

### Future perspectives

Stakeholders outlined strategies and proposals for improving OHC and IPC for older adults in HBC. These statements were analysed as a distinct main category. Three key areas emerged: framework conditions, education, and communication.

### Framework conditions

Stakeholders advocated for structural reforms to strengthen IPC. These included improved funding mechanisms, simplified cost-approval procedures, and extended billing options for PC: ‘*We usually bill through service packages, and the requirements set by the expert standard—not just that one, but other expert standards as well—actually call for a dedicated service package for oral hygiene in billing.’ (FG3_PP, Pos. 464, Speaker: PP12-PC)*.

Proposals also included mandatory cooperation agreements between dental practices and ambulatory care providers, as well as the integration of OHC into existing disease management programmes, such as a disease management programme for type 2 diabetes mellitus programme. For example, GPs could be encouraged to examine the oral cavity during scheduled visits and remind patients to attend regular dental appointments.

### Education

Stakeholders recommended targeted training: *‘Training specifically for oral health is probably needed in all professional groups, except maybe for dentists […]’ (EI_HÄ09, Pos. 110; Speaker: GP).* This included interprofessional education, extended curricula, and low-threshold educational materials (e.g. brochures and flyers) for PC and FC.

### Communication

The importance of effective and structured communication within IPC was strongly emphasised. Participants called for digital solutions, such as electronic patient records and secure messaging, to improve information flow and coordination. Local care networks were viewed as particularly beneficial for fostering direct exchange: *‘I wish we all had the same communication system […]. I really want to be the central point of contact as a general practitioner […].’* (EI_HÄ01, Pos. 130; speaker: GP).

In this context, PC were seen as key connectors in future care models due to their close and continuous contact with all stakeholder groups. Furthermore, regular patient conferences and interprofessional case discussions, complemented by the written exchange of information through referral forms and medical reports, were identified as potential measures for optimising communication pathways.

Across all domains, the findings illustrate that OHC for older adults in HBC is shaped by a complex interplay of individual, organisational, and structural factors. Barriers, such as limited coordination, unclear responsibilities, and restricted access, predominated stakeholder accounts, yet facilitators highlighted the importance of supportive networks, clear professional mandates, and proactive communication. These insights provide a foundation for developing targeted strategies to strengthen IPC and support oral health in this vulnerable population.

## Discussion

While several studies have examined OHC for older, care-dependent individuals in Germany [37–41], this is the first study to integrate the perspectives of all key stakeholder groups involved in HBC. Our findings underscore the complexity of oral health provision in HBC by revealing cross-cutting barriers and facilitators across different professions and care contexts, encompassing both daily oral health care and the utilisation of dental services. The results highlight three central themes: difficulties in maintaining daily oral hygiene, barriers to accessing of dental services, and restrained IPC within this setting.

Across all stakeholder groups, cognitive impairment, particularly dementia, emerged as the most significant challenge for maintaining oral hygiene in HBC. Affected older adults often lack the cognitive and functional capacity to perform daily oral hygiene independently, which contributes to deteriorating oral health. These observations are consistent with prior evidence, such as the systematic review by Delwel et al. [25], which highlights the impact of dementia on oral health status and care needs.

As older adults with dementia often struggle to communicate pain or discomfort related to oral health, this can hamper the timely recognition of dental problems and delay access to appropriate care. While this issue has been addressed primarily in inpatient settings [42, 43], our study shows that it is equally relevant in HBC, where approximately 70% of interviewed FC supported relatives with dementia. This reflects the widespread prevalence of dementia among care-dependent older adults in Germany [44], not a bias in sampling. In addition, poor oral hygiene is not limited to people with dementia: the 6th German Oral Health Study (DMS 6) [18] found that tooth brushing skills and oral hygiene behaviour are insufficient across all age groups, suggesting that cognitive impairments exacerbate an already widespread problem. This is supported by findings from Switzerland where Besimo and Besimo-Meyer [45] described substantial communication challenges when discussing oral hygiene with people living with dementia.

Oral hygiene is perceived as a highly intimate and private task, reinforcing the reluctance to accept external support and complicating its provision and its acceptance by adults in HBC. Brushing by third parties involves not only physical contact, but also interpersonal trust, cultural norms, and emotional boundaries [46]. Our results confirm that many older adults refuse oral care, especially when provided by unfamiliar caregivers-echoing previous findings on defiant behaviour during hygiene routines [16, 46]. In particular, PC, FC, and DA all emphasised that the oral cavity is experienced as a particularly private area, reinforcing the reluctance to accept external support.

Although the PC in our study acknowledged oral care as part of their responsibility, they frequently reported difficulties in delivering adequate oral hygiene due to time constraints. Ambulant care services bill for their provided services either through standardised service modules or individually agreed service models in Hamburg [47]. Oral care is bundled into broader care modules- such as morning or evening routines- that also include dressing, washing, and other tasks. This structural setup often limits the time available for individualised oral hygiene, particularly when care recipients require more attention due to cognitive or physical impairments. These challenges mirror findings from Göstemeyer et al. [16], who noted that time pressure is a widespread barrier among PC.

Oral care, particularly daily hygiene, was often perceived as a low priority by both caregivers and care recipients, and its importance appeared to decline with increasing care dependency. With the exception of dentists, all stakeholder groups in this study regarded dentistry as having a comparatively low priority within general medical care for older adults in HBC. Many professionals perceived dental care as holding lower status within the broader healthcare system, a view confirmed by dentists, who noted that oral health issues are frequently deprioritised by other healthcare providers. Communication, particularly regarding medication-related risk- is typically initiated by dental professionals themselves [48].

In addition, social factors such as lifestyle and socioeconomic status were perceived as influencing attitudes towards oral hygiene. These finding is consistent with Niesten et al. [49], who reported that oral care is often undervalued in the broader context of personal hygiene. To address this issue, all healthcare professionals involved in care for older adults should posses at least basic knowledge of geriatric oral health [50]. A recent systematic review demonstrated that targeted oral health interventions can effectively improve such knowledge [51].

Financial barriers were consistently identified across stakeholder groups as a major obstacle to accessing dental services. Older adults in need of HBC often avoided dental visits due to concerns about treatment costs, particularly when services exceeded standard health insurance coverage and required out-of-pocket payments. Our findings align with previous research [52], which likewise identified financial burden as a key barrier to dental service utilisation among older adults.

Stakeholders consistently emphasised the importance of a well-functioning support network in maintaining oral health among care-dependent older adults. Such networks, often composed of FC and PC, facilitate daily oral hygiene and help coordinate dental appointments. Participants reported that in the absence of a such support, dental visits tend to decline and oral health deteriorates. These findings are consistent with those of Burr et al. [53] who demonstrated that the presence of social support network increases the likelihood of dental services utilisation.

Our results also confirm that IPC in the OHC of older adults in HBC remains infrequent. Stakeholders identified poor communication and a lack of coordination as key barriers, with IPC often limited to acute situations rather than integrated into routine care. This aligns with previous research suggesting that the complexity of home-based nursing correlates with the perceived need for IPC [54]. Establishing standardised procedures and clearly defined roles, for example through structured cooperation between GPs and PC, in which GPs provide medical instructions and PC implement care, may support more continuous collaboration [55].

Barriers such as inadequate communication and fragmented information exchange were consistent with previous research [56, 57]. Professional caregivers also highlighted entrenched hierarchies as a persistent obstacle to equitable collaboration [56, 58]. Difficulties in contacting other professionals, due to unclear contact pathways, time constraints, or professional silos, further undermined cooperation, as also described by Meidert & Ballmer [57]. Additional barriers frequently reported in the literature include insufficient funding mechanisms, vague role definitions, and the absence of binding organisational frameworks to support structural IPC [24–26].

Furthermore, patients and their relatives were frequently positioned as informal intermediaries in the exchange of information between GPs and dentists. This indirect communication path was associated with information loss and increased administrative burden, particularly from the perspective of dental professionals. Our findings align with those of Huettig [48] and Sekanina et al. [54], who likewise described patients as default communication channels, a practice that hampers effective collaboration and leads to avoidable inefficiencies [57].

Despite these challenges, stakeholders identified several conditions that can faciliate effective IPC in the context of home-based OHC. Geographical proximity and personal familiarity between professionals, especially between GPs and dentists, were viewed as key enablers of collaboration. These observations are supported by previous research: both Gurtner and Wettstein [59] and Sekanina et al. [54] highlighted the positive influence of spatial closeness and established interpersonal relationships on effective cooperation.

Moreover, participants emphasised that regular exchange, supported by standardised procedures and shared care routines, can foster more structured and sustainable IPC. Such practices align with the interprofessional competencies outlined by Vaseghi et al. [60], which emphasise clear communication channels, well-defined responsibilities, and mutual trust as prerequisites for effective collaboration.

Taken together, these findings underscore the need for system-level strategies to strengthen communication structures and promote routine interprofessional exchange.

## Future perspectives

Looking ahead, stakeholders called for financial reforms to reduce the burden of OHC for older adults in HBC. They recommended full reimbursement for professional dental cleaning and coverage of transport costs, both of which were frequently cited as barriers to access. Participants also proposed shared financing models, such as pooled budgets or bundled payment between GPs, dentists, care organisations, and insurers, to support collaborative structures and reduce administrative hurdles. These ideas echo previous findings that highlight inadequate funding as a key barrier to IPC [37, 61].

Stakeholders further emphasised the need for innovative collaboration formats. Regular interprofessional case discussions were viewed as central to improving mutual understanding, coordination, and continuity of care. Structured dialogue, shared documentation, and digital communication tools were seen as practical means to foster more efficient IPC [62].

Targeted education and training across all professional groups were identified as critical to sustaining improvement in OHC. Stakeholders supported interprofessional teaching within undergraduate curricula, as well as joint continuing education for GPs and dentists to promote knowledge exchange and challenge professional boundaries, as emphasised by the WHO [63] and the Scientific Commission of the German Science and Humanities Council [64]. For PC, even brief training interventions were reported to enhance competence and willingness to collaborate [62, 65, 66]. In addition, FC and people in need of care expressed a desire for accessible, low-threshold information materials, though such resources remain underutilised in practice [67, 68].

In summary, the findings suggest three key areas for action:

- Support for caregivers: targeted training for FC and sufficient time and workflow integration for PC.
- Improved access: reimbursement policies covering transport and fair compensation for home visits.
- Strengthened IPC: standardised documentation, digital platforms, financial incentives, and clear role definitions.

### Strengths

The methodological quality of the study was appraised using the distinction between internal and external validity, as recommended by Kuckartz [33]. Internal validity, referring to the consistency and transparency of the analytical process, was supported by the use of a structured coding frame, investigator triangulation, and systematic documentation, including a supplementary table. The combination of focus groups and individual interviews enabled the exploration of shared experiences as well as individual nuances, enhancing the depth and credibility of findings.

Dependability and confirmability [33] were strengthened by involving multiple researchers throughout the analysis, maintaining an audit trail, and documenting decisions through reflexive memo-writing. Credibility of the findings was further supported by triangulation across methods and professional groups, as well as continuous peer debriefing and presentation of findings in regular stakeholder meetings twice a year.

The interdisciplinary backgrounds of the research team, spanning nursing science, physiotherapy, and health care management, contributed to analytical depth and reflexivity. The two-phase study design also allowed cross-validation of findings across settings and stakeholder groups, thereby enhancing analytical robustness. Thematic saturation [69] was considered to have been achieved, as no relevant themes emerged in the final individual interviews or focus groups.

### Limitations

External validity, referring to the transferability of the findings to other contexts, may be limited as the study was conducted exclusively in Hamburg. While the sample size was appropriate for a qualitative design, it does not allow generalisation. As is typical for qualitative research, the findings may reflect subjective experiences and perceptions, and the positionalities of the research team may have influenced interpretation despite efforts to ensure reflexivity.

Care-dependent older adults were not included in the qualitative component, as the focus was placed on PC and FC involved in OHC and on IPC among stakeholders. This limits direct insight into patients’ lived experiences; however, complementary quantitative data from a survey involving 1,622 care recipients (median age: 83.2 years) were published elsewhere [70], and clinical dental examinations were conducted in another part of the study [27].

Some degree of homogeneity within stakeholder groups, particularly among dentists and dental assistants, may have constrained the range of perspectives. The sampling strategy may have favoured participants with a pre-existing interest in the topic, introducing potential selection bias. Certain relevant actors, such as live-in caregivers or migrant care workers (often from Eastern European countries), were not included and were therefore not represented in the sample. Due to the non-personalised recruitment process, a response rate could not be calculated.

## Conclusion

While regional care models may differ across Germany and local pilot projects may have influenced stakeholder experiences, this study offers transferable insights into key challenges and strategies for improving OHC in HBC settings.

By integrating the perspectives of all major stakeholder groups, the study identified barriers and facilitators across three central domains:

1. Daily oral health care: Tasks related to oral hygiene are often supported or taken over by FC or PC, who bear most of the responsibility despite limited training and unclear guidance. Although PC generally receive basic instruction and follow care standards, they often lack time and resources to adequately integrate OHC into daily routines. Strengthening structural conditions, professional recognition, and targeted training for FC and PC is therefore essential.
2. Utilisation of dental services: Access to and utilisation of dental services are constrained by mobility constraints, financial burden, and insufficient reimbursement. Structural adjustments and equitable funding mechanisms are needed to improve access and uptake.
3. IPC: Fragmented care structures and limited communication between care providers hinder coordinated care. Standardised documentation, digital tools and financial incentives could strengthen cooperation and continuity of care.

Given demographic trends and the increasing complexity of care needs, these findings provide practical guidance for future health services planning and policy development. The InSEMaP study contributes a comprehensive foundation for further research on integrated OHC models in ambulatory and home-based care.

## Supplementary Information

Supplementary Material 1.

Supplementary Material 2.

Supplementary Material 3.

## Data Availability

The interview and focus group datasets generated and analysed during the current study are not publicly available due to an agreement with the participants on the confidentiality of the data. However, the data are available from the corresponding author on reasonable request.

## Abbreviations

DA: Dental assistant
FC: Family caregiver
GP: General practitioner
HBC: Home-based care
InSEMaP: Interaction of Systemic Morbidity and Oral Health in Ambulatory Patients in Need of Home Care
IPC: Interprofessional Collaboration
OHC: Oral Health Care
PC: Professional caregivers
QR: Qualitative research
