# Supplementary material for: Barriers, challenges, and facilitators in oral health care for older adults in home-based care: stakeholder perspectives from germany
Source: BMC Geriatr. 2026 Sep 10;26:1167. doi: 10.1186/s12877-026-08219-7 (PMC13576217; doi:10.1186/s12877-026-08219-7)
Supplement: Supplementary file 1 [file 12877_2026_8219_MOESM1_ESM.pdf]

File 1: Interview guide for individual interviews and homogenous focus groups

1. Introduction section

- Interviewer introduction, confidentiality, digital recording
- Please give me a little introduction on yourself.

2. Main questions

2.1 Part one: facilitating and hindering factors affecting oral health care

**Individual case vignettes as stimuli for each stakholder*

<u>professional and family caregivers:</u>

- Tell us about your experiences with the oral healthcare of elderly people in need of home-based care.
- What responsibilities do you undertake in the daily oral and dental care of elderly people in need of home-based care/ the person under your care?

<u>General practitioner, dentist and dental assistants:</u>

- How do you perceive the dental care of elderly dependent people in need of home-based car?
- How do you support older people in need of home-based care with their dental care?

<u>For all stakeholders:</u>

What challenges do you experience in the regular oral healthcare of older people in need of home-based care?

2.2 Part two: facilitating and hindering factors affecting utilisation of dental services

**Individual case vignettes as stimuli for each stakholder*

<u>professional and family caregivers:</u>

- Which responsibilities do you undertake to organize and coordinate dental services for elderly people in need of home-based care?

- How do you address the challenges of accessing dental services and meeting the specific needs of older people in need of home-based care?

General practitioner, dentist and dental assistants:

- What tasks do you consider spontaneously essential in the organisation and coordination of dental services for older people in need of home-based care?
- How do you address the challenges associated with the needs (and treatment) of people in need of home-based care in your practice?

Additionally for professional caregivers, dentists and dental assistants:

- There have been several legislative changes recently. Have you noticed any changes in dental care for older people in need of home-based care?

For all stakeholders:

- In your opinion, what are the main reasons that lead or have led to a demand for dental services from older people in need of home-based care?
- In your opinion, what are the main reasons that lead or have led older people in need of home-based care to a reduction or even discontinuation of dental services?

2.3 Part three: current status of interprofessional collaboration

Individual case vignettes as stimuli for each stakeholder

For all stakeholders:

- How do you perceive the collaboration and communication between you and the other stakeholders involved in oral health care?
- What experiences have you had with collaboration with other medical professions in oral health care?
- How well do you feel informed about dental/general medical conditions and treatments (including medication) that impact general medical/oral healthcare?
- How do you perceive the collaboration between general practitioners and dentists?

2.4 Part four: wishes and ideas for future interprofessional collaboration and improvement of oral health care.

For all stakeholders:

- If you could wish for something in the future to improve the collaboration between the stakeholders involved in the oral healthcare of older people in need of home-based care, what would it be?

3. Closure

For all stakeholders:

- Do you want to add something that we did not discuss yet?
- Acknowledgments
