# Supplementary material for: Barriers, challenges, and facilitators in oral health care for older adults in home-based care: stakeholder perspectives from germany
Source: BMC Geriatr. 2026 Sep 10;26:1167. doi: 10.1186/s12877-026-08219-7 (PMC13576217; doi:10.1186/s12877-026-08219-7)
Supplement: Supplementary file 2 [file 12877_2026_8219_MOESM2_ESM.pdf]

File 2: Interview guide for heterogeneous focus groups

1. **Introduction section**

- Interviewer introduction, confidentiality, digital recording
- Please give me a little introduction on yourself.

2. **Main questions**

2.1 Part one: Self-reflection on one's own role in the oral health care process of older people in need of home-based care

Could you please tell us what the responsibilities of your stakeholder group are, or what your stakeholder group feels responsible for in the oral healthcare of older people in need of home-based care?

2.2 Part two: Understanding the role of other stakeholder groups in the oral healthcare of older people in need of home-based care

- Could you please tell us how, as a representative of your stakeholder group, you perceive the responsibilities of the other stakeholders in the oral healthcare of older people in need of home-based care?
- Could you please tell us what expectations you, as a representative of your stakeholder group, have of the other stakeholders involved in the care process?

2.3 Part three: Future interprofessional collaboration

If you could wish for something in the future to improve the collaboration between the stakeholders involved in the oral healthcare of older people in need of home-based care, what would it be?

3. Closure

- Do you want to add something that we did not discuss yet?
- Acknowledgments
