# Supplementary material for: Barriers, challenges, and facilitators in oral health care for older adults in home-based care: stakeholder perspectives from germany
Source: BMC Geriatr. 2026 Sep 10;26:1167. doi: 10.1186/s12877-026-08219-7 (PMC13576217; doi:10.1186/s12877-026-08219-7)
Supplement: Supplementary file 3 [file 12877_2026_8219_MOESM3_ESM.pdf]

COREQ-Checklist

Topic	Item No.	Guide Questions/Description	Remarks	Reported on Page No.
**Domain 1: Research team and reflexivity**				
Personal characteristics				
Interviewer/ facilitator	1	Which author/s conducted the interview or focus group?	All focus group discussions were facilitated by AKoe or SP, with MH attending as a note taker.	p. 14
Credentials	2	What were the researcher's credentials? E.g. PhD, MD	SP: M.Sc.<br>MH: Student M.Sc.<br>AK: Student MPH	p. 15
Occupation	3	What was their occupation at the time of the study?	SP: female, research assistant, postgraduate student PhD, nursing science/ care management (M.Sc.)<br>MH: female, research assistant, postgraduate student Health Economics and Health Care Management (M.Sc.)<br>AK: female, research assistant, postgraduate student of Public Health (MPH).	p. 15
Gender	4	Was the researcher male or female?	All researchers are female.	p. 15
Experience and training	5	What experience or training did the researcher have?	SP and AKoe were experienced in qualitative methods and MH, who was new to qualitative research, received extensive training and close supervision.	p. 15
Relationship with participants				
Relationship established	6	Was a relationship established prior to study commencement?	Interviewers and participants were either unfamiliar or loosely acquainted, such as through professional associations.	p. 11
Participant knowledge of the interviewer	7	What did the participants know about the researcher? e.g. personal goals, reasons for doing the research	Prior to data collection, interviewers introduced themselves as project staff and provided both verbal and written information about the study.	p. 14
Interviewer characteristics	8	What characteristics were reported about the inter viewer/facilitator? e.g. Bias, assumptions, reasons	The characteristics of each author have been reported in the section titled "Researchers characteristics".	p. 15

		and interests in the research topic		

Domain 2: Study design
Theoretical framework

Methodological orientation and Theory	9	What methodological orientation was stated to underpin the study? e.g. grounded theory, discourse analysis, ethnography, phenomenology, content analysis	Data were analysed using the content-structuring method as described by Kuckartz, utilising both deductive categories from the interview guide and inductive categories emerging from the material.	p. 14/15

Participant selection

Sampling	10	How were participants selected? e.g. purposive, convenience, consecutive, snowball	Participant recruitment for both phases was carried out in cooperation with different organisations in Hamburg, including the Association of Statutory Dentists, the Association of General Practitioners, the Hamburg Care Society, and the Alliance for Caregivers.	p. 9-11
Method of approach	11	How were participants approached? e.g. face-to-face, telephone, mail, email	Organisations were initially contacted by phone to confirm their willingness to support the recruitment process. Subsequently, they were provided with tailored recruitment letters to be distributed inside the organisations. (…)<br>Participants for the second phase of data collection were predominantly contacted personally and were also provided with a recruitment letter.	p. 11-13
Sample size	12	How many participants were in the study?	A total of N=55 participants were recruited for the initial and subsequent phases of data collection. In the first phase, 17 individual interviews and six homogeneous focus groups with 29 participants were conducted (n=46). Additionally, nine participants took part in the second phase (heterogeneous focus groups).	p. 15
Non-participation	13	How many people refused to participate or dropped out? Reasons?	There were no dropouts between recruitment and data collection, and all participants gave written informed consent.	p. 14

Setting

Setting of data collection	14	Where was the data collected? e.g. home, clinic, workplace	Most of the focus groups (n=6) were conducted digitally via Cisco WebEx, a secure video conferencing tool that ensures confidentiality through password-protected, individual invitations. The homogenous focus group for family caregivers was held in presence to encourage participation. A total of two homogenous focus groups were conducted with professional caregivers; the second group was also held in presence, after the first group consisted of solely participants with management experience and the second group consisted exclusively of participants from a large outpatient care service. A total of n=17 individual interviews were audio-recorded, of which 16 were conducted by telephone and one in person at the participant's request.	p. 13/15-16
Presence of non-participants	15	Was anyone else present besides the participants and researchers?	No-one else was present besides the participants and the researchers. All focus group discussions were facilitated by AK or SP, with MH in attendance as a note taker.	p. 13-14
Description of sample	16	What are the important characteristics of the sample? e.g. demographic data, date	The important characteristics of the sample have been reported in the section titled “Sampling”.	p. 16, table 1

Data collection

Interview guide	17	Were questions, prompts, guides provided by the authors? Was it pilot tested?	The characteristics of the interview guide have been reported in the section “Method of data collection”; The focus group pretest included three professional caregivers. Based on their feedback, we revised the phrasing of key questions and adjusted the sequencing of discussion prompts.	p. 8-9, Supplementary file 1 and file 2
Repeat interviews	18	Were repeat interviews carried out? If yes, how many?	For PC, two groups were conducted due to heterogeneity in roles. The first PC group included only individuals in management roles, while the second consisted of staff from a large outpatient care organisation.	p.14-16

Audio/ visual recording	19	Did the research use audio or visual recording to collect the data?	The focus groups were conducted with audio recordings and postscript documentation created for each focus group. The individual interviews were conducted with the help of audio recordings.	p. 13
Field notes	20	Were field notes made during and/or after the interview or focus group?	The focus group interviews were documented with a postscript.	p. 14
Duration	21	What was the duration of the inter views or focus group?	The mean duration of focus groups was 97 minutes (range: 75-121), and individual interviews averaged 36 minutes (range: 17-57).	p. 16
Data saturation	22	Was data saturation discussed?	While sample sizes were pre-defined based on the project design, we considered thematic saturation as an analytical reference point. Thematic saturation was plausible achieved, as no relevant themes emerged in the final individual interviews or focus groups.	p.11/33
Transcripts returned	23	Were transcripts returned to participants for comment and/or correction?	The transcripts were not returned to participants for correction or comment.	p. 14

Domain 3: analysis and findings
Data analysis

Number of data coders	24	How many data coders coded the data?	Three data coders: MH, AKoe, SP	p. 14
Description of the coding tree	25	Did authors provide a description of the coding tree?	Main categories were defined a priori, while subcategories were generated during the analysis. All codes were based on meaningful units, ensuring that each segment was comprehensible on its own. After coding, categories were refined through internal discussions with research colleagues.	p. 16-17, table 2/table 3
Derivation of themes	26	Were themes identified in advance or derived from the data?	The Themes were defined a priori (based on the interview guide).	p. 14
Software	27	What software, if applicable, was used to manage the data?	MAXQDA software was used for data management.	p. 14
Participant checking	28	Did participants provide feedback on the findings?	No feedback of participants on the findings.	n/a

Reporting

Quotations presented	29	Were participant quotations presented to illustrate the themes/findings? Was each quotation identified? e.g. participant number	Yes, quotations were presented and identified by the interview code, the respondent's pseudonymisation code and the position in the interview.	p.18-25, table 3
Data and findings consistent	30	Was there consistency between the data presented and the findings?	Yes, there was consistency between the data presented and the findings.	p. 25-32
Clarity of major themes	31	Were major themes clearly presented in the findings?	Yes, major themes were clearly presented in the findings.	p. 25-32
Clarity of minor themes	32	Is there a description of diverse cases or discussion of minor themes?	No, there was no descriptions of the diverse cases or discussion of minor themes presented.	n/a
